# Time Series Analysis of Incidence Data of Influenza in Japan

**DOI:** 10.2188/jea.JE20090162

**Published:** 2011-01-05

**Authors:** Ayako Sumi, Ken-ichi Kamo, Norio Ohtomo, Keiji Mise, Nobumichi Kobayashi

**Affiliations:** 1 Department of Hygiene, Sapporo Medical University School of Medicine, Sapporo, Japan; 2 Department of Liberal Arts and Sciences, Sapporo Medical University, Sapporo, Japan; 3 Natural Energy Research Center Co. Ltd., Sapporo, Japan; 4 Center for Medical Education, Department of Admissions, Sapporo Medical University, Sapporo, Japan

**Keywords:** time series analysis, influenza, surveillance

## Abstract

**Background:**

Much effort has been expended on interpreting the mechanism of influenza epidemics, so as to better predict them. In addition to the obvious annual cycle of influenza epidemics, longer-term incidence patterns are present. These so-called interepidemic periods have long been a focus of epidemiology. However, there has been less investigation of the interepidemic period of influenza epidemics. In the present study, we used spectral analysis of influenza morbidity records to indentify the interepidemic period of influenza epidemics in Japan.

**Methods:**

We used time series data of the monthly incidence of influenza in Japan from January 1948 through December 1998. To evaluate the incidence data, we conducted maximum entropy method (MEM) spectral analysis, which is useful in investigating the periodicities of shorter time series, such as that of the incidence data used in the present study. We also conducted a segment time series analysis and obtained a 3-dimensional spectral array.

**Results:**

Based on the results of power spectral density (PSD) obtained from MEM spectral analysis, we identified 3 periodic modes as the interepidemic periods of the incidence data. Segment time series analysis revealed that the amount of amplitude of the interepidemic periods increased during the occurrence of influenza pandemics and decreased when vaccine programs were introduced.

**Conclusions:**

The findings suggest that the temporal behavior of the interepidemic periods of influenza epidemics is correlated with the magnitude of cross-reactive immune responses.

## INTRODUCTION

Influenza is an acute contagious disease caused by a virus. Three influenza pandemics have occurred during the 20th century (in 1918, 1957, and 1968),^1^ each of which resulted in more than a million deaths over a short period of time. Recently, the ongoing worldwide spread of the H1N1 influenza virus has increased concerns of a new human influenza. Accordingly, much effort to prevent influenza has been expended on infectious disease surveillance, vaccinations, and various theoretical and experimental research.^1^ Of these, there has been great interest in developing approaches to elucidate the structure of temporal variations in influenza epidemics, using mortality and morbidity records of the disease.^2^^–^^5^

Time series analysis has been widely used in epidemiology to predict epidemics of infectious diseases, including influenza.^6^^–^^13^ Since 1970, the World Health Organization has requested influenza mortality data from selected representative nations, in order to produce estimates of the worldwide impact of the disease, and attempts have been made to estimate excess mortality, which is useful as an early quantitative index of an influenza epidemic.^6^ Choi and Thacker^7^ demonstrated that time series analysis was useful in estimating excess mortality during 8 influenza epidemics occurring in the United States from 1967 to 1978.

Recently, there has been considerable interest in interpreting the mechanism of influenza epidemics.^10^^–^^17^ Because influenza shows annual cycles in prevalence, it is reasonable to investigate seasonal forcing, ie, the extent to which external force can amplify the oscillation of annual cycles. Urashima et al^12^ explained oscillation in the number of influenza cases in Tokyo in terms of climate, namely, temperature, humidity, and other factors. Aside from the obvious annual cycle of influenza epidemics, mortality records reveal longer-term incidence patterns, as are observed in common childhood diseases such as measles.^18^^–^^20^ This longer-term period, which is referred to as the interepidemic period, has long been a focus in epidemiology.^21^ The interepidemic period represents the amount of time required to accumulate a cohort of susceptible individuals that is sufficiently large to allow pathogens of infectious diseases to efficiently spread over a community once the pathogen is introduced from outside the community. For measles, Anderson et al^18^ used time series analysis of incidence data in England and Wales to reveal a biennial cycle in the interepidemic period and to show that vaccination programs alter this interepidemic period. Aron and Schwartz^22^ interpreted the biennial cycle for measles epidemics as the effect of seasonal variation in contact rate among school-aged children, based on the results of the Susceptible/Exposed/Infective/Recovered (SEIR) model, which is a well-known mathematical model of infectious disease epidemics.^20^^–^^22^

With respect to influenza, Clegg^23^ investigated the interepidemic period by using disease mortality records in Scotland. However, in the mortality records of influenza, precise temporal incidence patterns can be masked by the statistically noisy process of inferring flu incidence from pneumonia and influenza deaths.^24^ Thus, there is a need for a more detailed investigation of the interepidemic period in influenza morbidity data. As Cliff et al^25^ point out, “in epidemiology, long-term morbidity series are of interest where there is a need to establish historical trends in disease incidence.” In addition, the previously accepted model of the epidemic behavior of influenza, in which a new influenza A subtype or “pandemic strain” would appear at a 10- to 14-year interval, is no longer tenable.^1^

Regarding influenza epidemics in Japan, studies have used influenza morbidity data to investigate the temporal features of the epidemics with respect to prediction analysis,^9^ correlation of the epidemics with weather conditions,^12^^,^^26^ and geographic movement of the epidemics^27^; however, the interepidemic period in morbidity data has not been investigated in detail. In Japan, monthly morbidity records of influenza were collected during 1948–1998, which encompasses the pandemics of 1957, 1968, and 1977. Using time series analysis of these long-term data, we investigated the interepidemic period in influenza morbidity records.

## METHODS

### Data source

In Japan, the system of influenza reporting detailed in the Communicable Disease Prevention Law was used during 1948–1998. The law required physicians to report all cases of clinically diagnosed influenza to a nearby health center within 24 hours. The health centers transferred these reports to the local governments and to the Ministry of Health and Welfare. Influenza morbidity data were regularly reported to the Secretariat of the Ministry of Health and Welfare and published in an annual periodical, *Statistics on Communicable Diseases*, during 1948–1998.^28^ Since 1999, the Infectious Diseases Control Law has been in force, and influenza epidemics are now monitored and reported under a new system.

In the present study, we used time series data on influenza morbidity reported in *Statistics on Communicable Diseases in Japan* during 1948–1998.^28^ The time series data represent the monthly number of reported influenza cases per 100 000 population from January 1948 through December 1998 (612 data points). This incidence data is unique in Japan in that it encompasses the pandemics of 1957, 1968, and 1977.

### Data analysis

The present study uses spectral analysis based on the maximum entropy method (MEM) in the frequency domain and a nonlinear least squares method (LSM) in the time domain. The present procedure comprised 3 steps (I–III). In step I, we preprocessed the incidence data to permit detection of the interepidemic period of the data in step II and to investigate the temporal behavior of the interepidemic period of the data in step III.

#### Step I: Preparing time series data for analysis

The preprocessing of the incidence data was conducted using the following 3 procedures:

(1) Equal sampling time intervals were chosen, missing data were compensated for, outliers were corrected, and logarithmic transformation was performed, if necessary.

(2) To determine the long-term trend of the incidence data, we performed MEM spectral analysis, which is a type of time series analysis in the epidemiology of infectious diseases.^6^^,^^29^^,^^30^ The spectral analysis produces a power spectral density (PSD), from which we can obtain power representing the amount of amplitude of the incidence data at each frequency^31^ (note the reciprocal relationship between the scales for frequency and period). A large magnitude for power at a frequency of 0.25 (1/year), for example, would indicate that a large portion of the amount of amplitude of the incidence data is expressed as a wave that repeats itself every 4 years. MEM spectral analysis is useful to investigate periodicities of short time series, such as the infectious disease surveillance data used in the present study.^32^^–^^36^ The formulation of MEM-PSD is described in Appendix.

(3) The long-term trend was calculated using the least squares fitting (LSF) method with the period obtained from the MEM-PSD. This trend was then removed by subtracting the LSF curve from the data, thereby yielding the residual time series data. The formulation of the LSF curve is described in Appendix.

#### Step II: Assignment of interepidemic periods of influenza epidemics

MEM spectral analysis of the residual time series data was used to identify interepidemic periods.

#### Step III: Segment time series analysis

To further investigate the interepidemic period of influenza epidemics, we performed segment time series analysis, which has been widely used in fields such as medical and biological science, as well as in the physical sciences and engineering.^19^^,^^32^^,^^37^^–^^39^

## RESULTS

### Temporal variation in influenza incidence data

**Figure 1.**
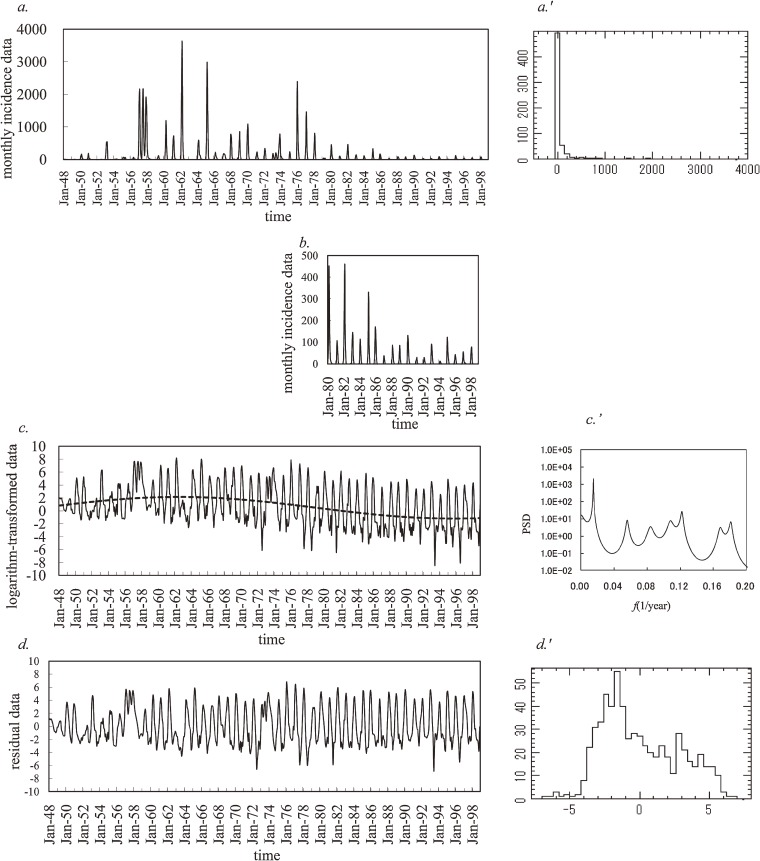
Monthly incidence data for influenza in Japan (1948 to 1998). *a*. the original data, *a*′. histogram of the original data, *b*. enlargement of the original data during 1980–1998, *c*. log-transformation of the original data (solid line) and the optimum least squares fitting (LSF) curve (dashed line), *c*′. Maximum entropy method power spectral density of the log-transformed data in the low frequency range (*f* < 0.2), *d*. residual data obtained by subtracting the LSF curve from the log-transformed data, and *d*′. histogram of the residual data.

Monthly incidence data on influenza in Japan during the period from January 1948 through December 1998 are plotted in Figure 1*a*. The major peak in 1957 represents the country’s first exposure to the H2N2 subtype, which caused the 1957 Asian flu pandemic. The quite extensive outbreaks in 1962 and 1965 were due to H2N2 variants that arose from antigenic drift. The next major peaks correspond with the 1968/1969 pandemic of Hong Kong flu, a new subtype (H3N2). The sporadic winter outbreaks during the 1970s were due to successive H3N2 variants, again arising from antigenic drift. In 1977, the H1N1 subtype re-emerged, after being totally absent since the 1950s, and caused the Russian flu. Peak incidence has been lower since the Russian flu; however, a closer view of the incidence data from 1980 to 1998 (Figure 1*b*) indicates that an annual cycle still exists, with a large peak in winter months. In Figure 1*b*, the 1980 outbreak involved a mixture of H3N2 and H1N1. Since then, mixed epidemics have been frequent during the 1980s and 1990s, and strains of the less virulent type B have been prominent. In addition, it is important to note that a special program to promote influenza vaccination among school children was started in 1962, and mass vaccination of school children was conducted from 1976 to 1994 under the Preventive Vaccination Law. Since 1994, influenza vaccination has been voluntary.^40^

### Preparing the incidence data for analysis

We take the incidence data *x*(*t*) (*t*: time) to represent discrete values at *t* = *k*Δ*t* (*k* = 1, 2, 3,…, *N*) where Δ*t* is the time interval and *N* is the length of the time series (in the present study, Δ*t* = 1 month and *N* = 612).

Figure 1*a*′ shows the frequency histogram for the incidence data. This histogram differs from the normal distribution required for conventional spectral analysis. Then, we performed logarithmic transformation of the incidence data (Figure 1*a*), which had 122 zero values. Some of these zero values were rounded zeros (ie, with a magnitude less than half of the unit employed); others were absolute zeros (ie, magnitude zero). To logarithmically transform incidence data with zero values, we added small, positive, random noise from a uniform (0, 0.1) distribution to the original incidence data (Figure 1*a*).^41^ Next, we multiplied a constant belonging (0, 1) to the time series data obtained in the first procedure. This constant is set so that the mean value of the time series data is equal to that of the original incidence data. We confirmed that there was no significant difference between the standard deviation of the original incidence data and that of the time series data obtained in the second procedure. Thus, we ensured that there would be no significant difference between the original incidence data and the adjusted time series data. Then, for the time series data thus obtained, we conducted the logarithmic transformation. In the log-transformed data shown in Figure 1*c*, the spikiness of the incidence observed in the original data is reduced and a long-term decreasing trend is evident.

In order to remove the long-term trend in the log-transformed data in Figure 1*c*, MEM-PSD, *P*( *f* ) ( *f* : frequency), for the log-transformed data was calculated. The PSD ( *f* ≤ 0.2) is displayed in Figure 1*c*′ (unit of *f* : 1/year). The longest period appears as a prominent peak at the position of the 66.7-year period. Using this 66.7-year period, we modeled the long-term trend in influenza epidemics by calculating the least squares fitting (LSF) curve for all the log-transformed data (Figure 1*c*). The formulation of the LSF curve is described in the Appendix. The LSF curve obtained (Figure 1*c*) accurately expressed the long-term trend in the log-transformed data. The LSF curve peaked in 1962, when the special influenza vaccination program began.

We then removed the LSF curve from the log-transformed data (Figure 1*c*) and the residual time series data were obtained, as shown in Figure 1*d*. The frequency histogram for these residual data is shown in Figure 1*d*′; it approximates the normal distribution required for conventional spectral analysis. Normality of distribution was assessed using the χ^2^ fitting test, and the null hypothesis was not rejected (*P* = 0.79).

### Identification of interepidemic periods of influenza epidemics

**Figure 2.**
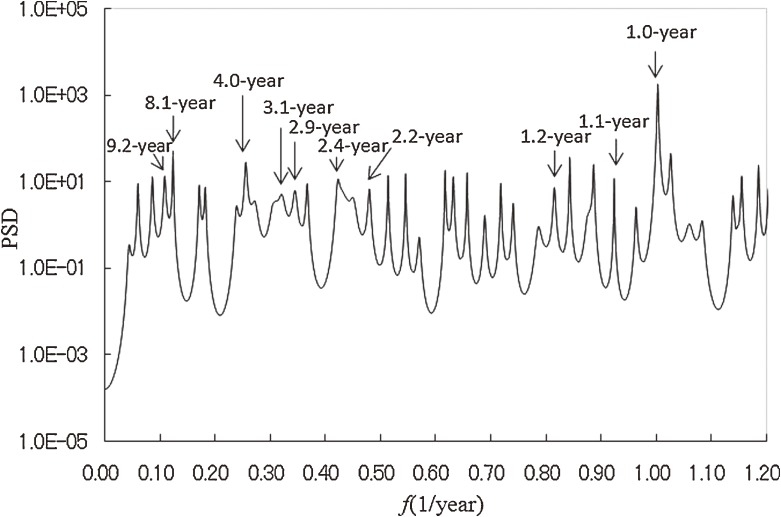
Maximum entropy method power spectral density (MEM-PSD) of the residual data in the low frequency range (*f* < 1.2).

To identify interepidemic periods in the incidence data, we conducted MEM spectral analysis. MEM-PSD was calculated for the residual data in Figure 1*d*, and the semi-log plot of the PSD ( *f* ≤ 1.2) is shown in Figure 2. The most prominent spectral peak is at *f* = 1.0 (= *f*_1_), which corresponds to a 1-year period, ie, the seasonal cycle of influenza epidemics in Japan. Ten peak spectral frequency modes were selected and are shown in the Table, with corresponding periods and powers. The 4.0-, 3.1-, and 2.4-year spectral peaks have relatively large powers, which indicates that they are the interepidemic periods of influenza epidemics in Japan.

**Table.** Characteristics of the 9 dominant spectral peaks shown in Figure 2

Frequency (1/year)	Period (years)	Power
0.11	9.2	0.06
0.12	8.1	0.09
0.25	4.0	0.16
0.32	3.1	0.12
0.34	2.9	0.07
0.42	2.4	0.16
0.45	2.2	0.06
0.84	1.2	0.07
0.89	1.1	0.10
1.00	1.0	4.28

### Segment time series analysis

**Figure 3.**
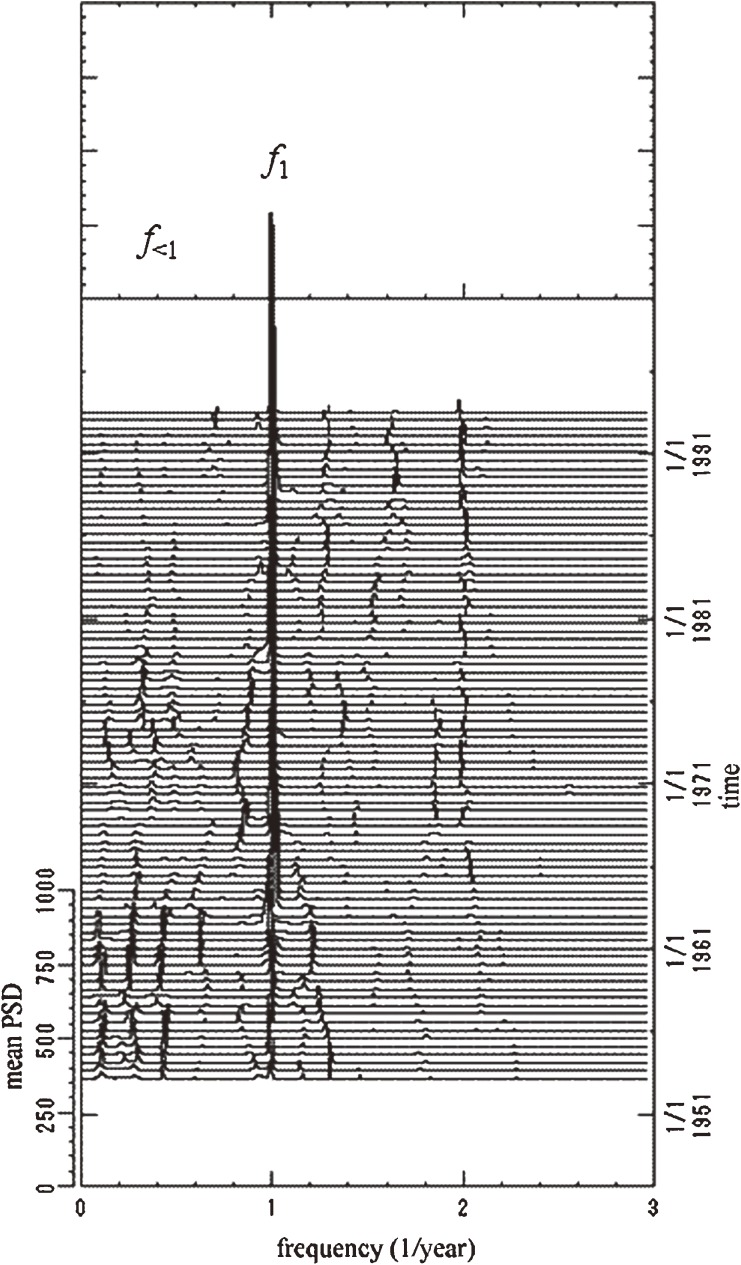
Three-dimensional spectral array of the residual data. PSD, power spectral density.

In the segment time series analysis, time series data were divided into multiple segments, and MEM-PSD was calculated for each segment. In this study, the residual data (Figure 1*d*) were divided into a subseries of 83: each segment had a time range of 120 months (10 years), which encompasses the longest dominant period, 9.2 years, for all the residual data, as shown in the Table; there is a 6-month delay at the beginning of the range. The PSDs thus obtained are shown as a 3-dimensional spectral array in Figure 3, in which frequency is represented on the horizontal axis and time on the perpendicular axis, running from bottom to top.

In this 3-dimensional spectral array, spectral lines at frequency *f* = 1.0 (= *f*_1_) corresponding to a 1-year period are visible as a fine array over the entire time range. This indicates that the influenza epidemics had a periodicity of 1 year. A notable feature in Figure 3 is that the spectral lines of *f*_<1_, which denotes a frequency lower than *f*_1_, vary in intensity. That is, the spectral lines of *f*_<1_ are clearly visible before 1963 and almost disappear during the period 1963–1971. They become again visible during 1971–1976, and, thereafter, become extremely weak. This is because the amount of amplitude of the interepidemic periods at frequency range *f*_<1_ changes over time, in contrast to *f*_1_.

**Figure 4.**
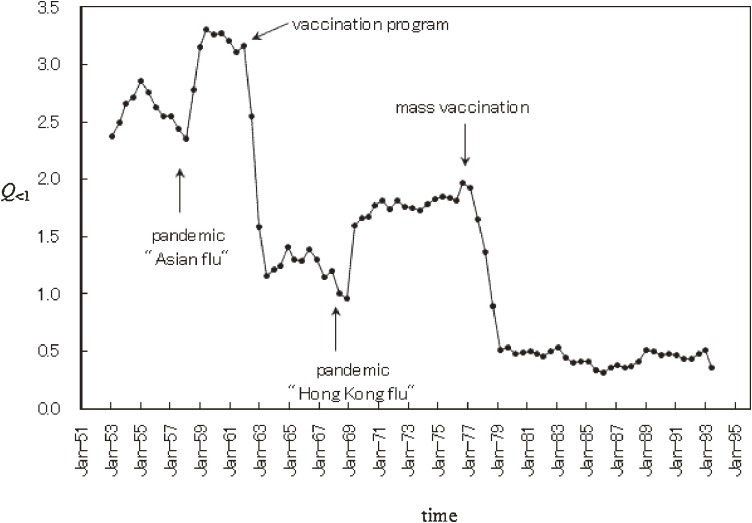
Temporal variation in the power of *f*_<1_ modes (*Q*_<1_).

To investigate the temporal behavior of the spectral lines at *f*_<1_, we calculated the power of *f*_<1_ modes in each segment, a value that was labeled *Q*_<1_. This is a measure of the amount of amplitude in residual data expressed by periodic modes longer than 1 year. We obtained *Q*_<1_ by integrating the PSD over the frequency range 0.05 to 0.8 (which corresponds to 1.25 to 20 years). The results are shown in Figure 4. The curve for *Q*_<1_ declines sharply in 1962 and 1977, and increases steeply in 1958 and 1969. These decreases in *Q*_<1_ in 1962 and 1977 occurred when vaccination programs were introduced in 1962 and 1976. The increases in *Q*_<1_ in 1958 and 1969 occurred after pandemics in 1957 and 1968. Thus, it appears that *Q*_<1_, ie, the amount of amplitude of interepidemic periods, increased during influenza pandemics and decreased at the start of vaccine programs.

## DISCUSSION

A significant result of the present study of influenza epidemics in Japan is that MEM spectral analysis enabled us to identify multiple periodicities in the interepidemic period of the disease epidemics (4.0-, 3.1-, and 2.4-year periods). This finding deserves special attention in light of the link between the temporal pattern of epidemics and the evolution of the influenza virus. Of the 3 types of influenza virus (A, B, and C), type A is epidemiologically the most important in humans, since influenza A virus undergoes 2 types of change, known as antigenic shift and antigenic drift. Antigenic shift gives rise to a novel subtype that can result in severe pandemics, eg, the Spanish flu of 1918 and the Hong Kong flu of 1968. Between pandemics, a succession of strains of the same subtype arise by antigenic drift and, with passing time, partial immunity—referred to as cross-reactive immunity—is conferred upon a host already infected by another strain of the same subtype. Plotkin et al^42^ reported that the average duration of cross-reactive immunity in influenza A virus varies between 2 and 5 years. Thus, the 4.0-, 3.1-, and 2.4-year interepidemic periods of influenza epidemics in the present study might correspond to the average duration of cross-reactive immunity.

In addition, the present results in conjunction with those of Plotkin et al^42^ suggest that the temporal behavior of *Q*_<1_ is correlated with the magnitude of cross-reactive immune response, ie, the increases in the magnitude of *Q*_<1_ in 1958 and 1969 might represent cross-reactive immune responses stimulated by the emergence of novel subtypes in 1957 (H2N2) and 1968 (H3N2), respectively. The decreases in *Q*_<1_ in 1962 and 1977 (Figure 4) might have resulted from declines in the magnitude of cross-reactive immune response, because the introduction of vaccine programs (in 1962 and 1976) played an important role in strain-specific immune response.

A campaign against vaccination began in the late 1980s due to concerns regarding the effectiveness of the Japanese vaccine program.^40^ In contrast to reports alleging that the influenza vaccine had little or no effectiveness, some studies indicated that the influenza vaccine was indeed effective in Japan.^43^^–^^45^ Sugiura et al^43^ conducted a randomized, controlled study of high school students during the 1968–1969 flu season and found that vaccine effectiveness against serologically confirmed infection was 80% (*P* < 0.001) for A (H3) and 43% (*P* < 0.01) for B. Based on reports of the effectiveness of the influenza vaccine in Japan,^43^^–^^45^ it is possible that the introduction of the influenza vaccine program had an effect on the interepidemic periods of the disease epidemics, as was the case with measles,^18^^,^^19^ and resulted in decreases in *Q*_<1_ in 1962 and 1977.

Many studies have used conventional time series analysis—including moving averages, multiple regression analysis, and an autoregressive (AR) model—to investigate the periodic structure of incidence data for infectious diseases.^6^^–^^13^ Quénel and Dab^11^ modeled the weekly incidences of influenza in France by using the seasonal autoregressive-integrated moving average (SARIMA) model and developed epidemic criteria. Kakehashi et al^9^ decomposed part of the incidence data used in the present study (Figure 1*a*) into a seasonal component, a quadratic trend, and an AR process. Such conventional time series analysis is useful in obtaining a model that fits well to time series data; however, this method has limitations in interpreting multiple periodic structures in time series data.^32^ Thus, in previous work,^9^ the interepidemic period of influenza epidemics in Japan was defined as an interval between major epidemics of the disease, similar to the seasonal cycle. In the present study, however, MEM spectral analysis enabled us to identify multiple periodicities for the interepidemic period of influenza epidemics (4.0-, 3.1-, and 2.4-year periods).

Recently, wavelet transform analysis has been used in time series analysis of incidence data for infectious diseases^46^; however, it sacrifices accuracy of frequency resolution for more precise time resolution.^47^ In addition, wavelet transform analysis requires a long time series.^46^ In contrast, the present method based on MEM spectral analysis enabled us to identify periodicities in shorter time series with a high degree of frequency resolution.^32^^,^^48^ In particular, the 3-dimensional spectral array obtained from segment time series analysis of short-term data revealed that periodic structures of the interepidemic period experience temporal change.

In general, biological phenomena are both nonstationary and nonlinear and transit from one state to another in a complicated manner. It can be said that the periodic structures of epidemics of infectious diseases, including influenza, change over time, based on the results obtained in the present study. To interpret temporal patterns in epidemics of infectious diseases, several mathematical models have been constructed using the theory of population dynamics of infectious diseases.^24^^,^^49^^–^^51^ These models have become invaluable management tools for epidemiologists who seek to interpret the mechanisms of epidemics. For influenza, work on mathematical models based on the theory of population dynamics has paralleled recent developments in molecular biology and computation, resulting in remarkable phylogenetic reconstructions of the evolution of the influenza virus.^42^ The link between population dynamics and viral evolution is central to the design of vaccines for influenza, although this connection is not well understood,^52^ perhaps because the conventional time series analyses used in studies of population dynamics deals exclusively with overall incidence data,^7^^–^^13^ which are subject to temporal variation. It is preferable to deal with shorter time series segments in segment time series analysis, as in the present study.

Regarding the logarithmic transformation of incidence data with a value of zero, we added small, positive, random noise with a uniform (0, 0.1) distribution to the original incidence data, as shown in Figure 1*a*.^41^ We also tried 2 alternative methods: (1) the addition of constants of 10^−4^%, 10^−3^%, and 10^−2^% relative to the largest values in the original incidence data (0.001, 0.01, and 0.1, respectively) and (2) the method used by Kakehashi et al,^9^ who analyzed part of the original incidence data analyzed in the present study (Figure 1*a*). As compared with the use of positive, random noise, these alternative methods yielded the same 4.0-, 3.1-, and 2.4-year periodic modes and the same temporal pattern of *Q*_<1_ shown in Figure 4. In addition, the frequency histogram for the data used in the present study more closely approximated the normal distribution required for conventional spectral analysis than did the distributions obtained using the 2 alternative methods. As a result, in the present study, we used the incidence data with small, positive, random noise.

In conclusion, our analysis of influenza epidemics in Japan indicates that there were multiple periodic modes (4.0, 3.1, and 2.4 years) for the interepidemic period in incidence data and that the periodic structure of the interepidemic period changes over time. We hypothesize that the temporal behavior of interepidemic periods of influenza epidemics correlates with the magnitude of cross-reactive immune response. Thus, effective long-term influenza control programs in Japan will require continued investigation of the interepidemic periods of disease epidemics, as well as careful examination of viral strains.

## APPENDIX

### MEM-PSD

The MEM-PSD for analysis of time series data, with an equal sampling interval Δ*t* (1 month in the present study), can be calculated as

$$p(f)=\frac{P_{m}\Delta t}{{\left|1+\sum_{k=-m}^{m}\gamma_{m,k}\exp[-i2\pi fk\Delta t]\right|}^{2}},$$ (A.1)

where *P_m_* is the output power of a prediction-error filter of order *m* and γ*_m_*_,_*_k_* is the corresponding filter coefficient, *m* = 0, 1, 2,…, *M* (*M* is the optimum filter order). The method for determining *M* is described in §5.1 in reference ^36^. *P_m_* and γ*_m_*_,_*_k_* are determined by solving the Yule-Walker equations with the use of Burg’s procedure.

### LSF calculation

LSF calculations are performed by using a suitable function

$$x(t)=a_{0}+\sum_{n=1}^{N_{p}}\{a_{n}\sin(2\pi f_{n}t)+b_{n}\cos(2\pi f_{n}t)\},$$ (A.2)

which is calculated using the least squares method (LSM) for *x*(*t*) with unknown parameters *f_n_*, *a*_0_, *a_n_*, and *b_n_* (*n* = 1, 2,…, *N_p_*), where *f_n_* (= 1/*T_n_*, *T_n_*: its period) is the frequency of the *n*th periodic component, *a*_0_ is a constant representing the average value of the time series, *a_n_* and *b_n_* are the amounts of amplitude of the *n*th periodic component, and *N_p_* is the total number of components. The periodic function (eq [Disp-formula eA.2]) can be determined using nonlinear LSM. Linearization of this nonlinearity is achieved by using the periods *T_n_* estimated by MEM spectral analysis.
